# Novel Synthesis of Phytosterol Ferulate Using Acidic Ionic Liquids as a Catalyst and Its Hypolipidemic Activity

**DOI:** 10.1021/acs.jafc.3c09148

**Published:** 2024-01-22

**Authors:** Wen-Sen He, Liying Zhao, Haonan Yang, Jiaxin Rui, Jie Li, Zhen-Yu Chen

**Affiliations:** † School of Food and Biological Engineering, Jiangsu University, 301 Xuefu Road, Zhenjiang, Jiangsu 212013, China; ‡ School of Life Sciences, The Chinese University of Hong Kong, Shatin ,Hong Kong, China

**Keywords:** oryzanol, phytosterol ferulate, acidic ionic liquid, esterification, lipid-lowering, cholesterol-lowering

## Abstract

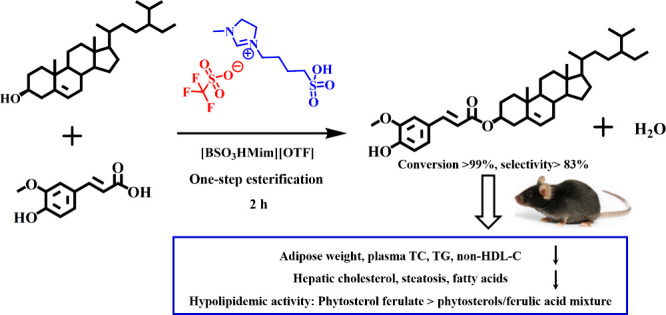

Phytosterol ferulate (PF) is quantitively low in rice, corn, wheat, oats, barley, and millet, but it is potentially effective in reducing plasma lipids. In this study, PF was synthesized for the first time using acidic ionic liquids as a catalyst. The product was purified, characterized using Fourier transform infrared, mass spectroscopy, and nuclear magnetic resonance, and ultimately confirmed as the desired PF compound. The conversion of phytosterol surpassed an impressive 99% within just 2 h, with a selectivity for PF exceeding 83%. Plasma lipid-lowering activity of PF was further investigated by using C57BL/6J mice fed a high-fat diet as a model. Supplementation of 0.5% PF into diet resulted in significant reductions in plasma total cholesterol, triacylglycerols, and nonhigh-density lipoprotein cholesterol by 13.7, 16.9, and 46.3%, respectively. This was accompanied by 55.8 and 36.3% reductions in hepatic cholesterol and total lipids, respectively, and a 22.9% increase in fecal cholesterol excretion. Interestingly, PF demonstrated a higher lipid-lowering activity than that of its substrates, a physical mixture of phytosterols and ferulic acid. In conclusion, an efficient synthesis of PF was achieved for the first time, and PF had the great potential to be developed as a lipid-lowering dietary supplement.

## Introduction

Cardiovascular disease (CVD) is the number one killer in the world.^1^ Hypercholesterolemia is a significant risk factor for CVD.^2^ Although drugs such as statins and ezetimibe are currently available to reduce the blood cholesterol level, they frequently entail a range of adverse effects. Phytosterols and their derivatives have garnered significant attention for their exceptional cholesterol-lowering properties.^3−5^ Among these compounds, phytosterol ferulate (PF) stands out as a derivative that combines the biological activities of both phytosterols and ferulic acid. While rice, corn, wheat, oats, barley, and millet are natural sources of PF, their quantity in these grains is relatively low.^6−10^ Currently, the major natural source of PF is oryzanol derived from rice bran or rice bran oil, but its content is below 25%.^11^ Extracting PF from oryzanol is a challenging task due to its structural resemblance to the primary component of oryzanol, triterpenol ferulate. To date, no effective method for isolating PF has been reported in the literature, highlighting the need to develop an efficient method for synthesizing this compound.

An early study employed three-step chemical reactions to synthesize phytosterol ferulate; however, it was plagued by multiple reactions and purification steps with a low conversion rate and a challenge in product separation.^12^ Subsequently, a method of two-step chemical-enzymatic reactions was introduced for the synthesis of phytosterol ferulate.^13^ In the first step, with mercuric acetate as a catalyst, the yield of vinyl ferulate was only 46% after 12 h of reaction. In the second step, using *Candida rugosa* lipase as a catalyst, the reaction required 10 days to complete. More recently, stand-alone enzymatic routes have been employed.^14^ However, these routes have been hindered by having a low conversion rate (below 55%) with long reaction times (up to 120 h) and a higher cost. Despite numerous attempts, an efficient synthesis for PF has not been developed to date.

Acidic ionic liquids (ILs), a type of low-temperature molten salt composed of anions and cations, have recently been used as catalysts for esterification and transesterification reactions due to their environmentally friendly, safe, and efficient characteristics.^15,16^ We have previously used a range of acidic ILs including 1-butylsulfonate-3-methylimidazolium hydrogen sulfate ([BSO_3_HMim]HSO_4_), 1-butylsulfonic-3-methylimidazolium tosylate ([BSO_3_HMIM]TS), and 1-butylsulfonic-3-methylimidazolium trifluoromethanesulfonate ([BSO_3_HMim]OTF) to synthesize hydrophilic phytosterol derivatives and phytosterol linolenate with conversion rates of 91% or higher,^17,18^ indicating that such acidic ILs can successfully catalyze the esterification of phytosterols. Furthermore, several acidic ILs such as [BSO_3_HMIM]TS and [BSO_3_HMim]HSO_4_ can serve as catalysts for the esterification of ferulic or caffeic acids with glycerol.^19,20^ These studies imply the feasibility of acidic ILs in catalyzing the esterification of phytosterols with phenolic acid.

Oryzanol is a mixture of ferulic acid esters of phytosterols and triterpenoids.^21,22^ As one component in oryzanol, PF is quantitively minor, and its cholesterol-lowering activity has not been explored. The present study was the first time to report an efficient method in the synthesis of PF using acidic ILs as a catalyst and explore its lipid-lowering potency in C57BL/6J mice.

## Materials and Methods

### Materials

Ferulic acid (purity >99%) was purchased from J&K Scientific Ltd. (Shanghai, China). β-Sitosterol (purity >95%) and phytosterols (54.5% sitosterol, 15.9% stigmasterol, and 27.3% campesterol) were provided by Sigma-Aldrich Co., Ltd. (Shanghai, China). All ILs (purity >99%) were obtained from Shanghai Chengjie Chemical Co., Ltd. (Shanghai, China). *n*-Hexane, methanol, and *iso*-propanol used for high-performance liquid chromatography (HPLC) analysis were of chromatographic grade and purchased from Tedia Co., Ltd. (Shanghai, China). Other reagents were of analytical grade and were obtained from Sinopharm Chemical Reagent Co., Ltd. (Shanghai, China).

### IL-Catalyzed Synthesis of Phytosterol Ferulate

β-Sitosterol was selected as a representative of phytosterols due to its high abundance in nature. β-Sitosterol (0.02–0.10 g), ferulic acid (0.03–0.09 g), the chosen ILs (0.0015–0.0110 g), and toluene (5 mL) were sequentially added into a 25 mL round-bottom flask. The flask was subsequently fixed in a heating oil bath equipped with a magnetic stirring apparatus. The reaction commenced when the temperature reached the setting value (80–120 °C) under condensate reflux and lasted for 0.5–4 h. During the reaction period, 0.2 mL of the reaction sample was periodically sampled and extracted for thin-layer chromatography (TLC) and HPLC analyses. Each reaction was repeated at least three times.

### Product Purification by Silica Gel Column Chromatography

Following the reaction, 8 mL of distilled water was added into the round-bottom flask for full extraction, and the sample in the toluene layer was collected and evaporated in a rotary evaporator. These samples were then separated on a silica gel column (1.2 × 100 cm) using petroleum ether/ethyl acetate (4:1, v/v) as the eluent. The eluate was collected in different test tubes and monitored by TLC analysis. Eventually, the samples in the tubes containing only the product were collected followed by removal of the solvent with a rotary evaporator. The byproduct was obtained using the same method.

### TLC Analysis

Approximately 10 μL of the sample was evenly spotted onto a preactivated TLC plate. The TLC plate was then developed using petroleum ether/ethyl acetate (4:1, v/v) as the developing agent and colored using iodine steam as the coloring reagent.

### HPLC Analysis

The quantitative analysis of the product and its purity detection was performed on an LC-20AB HPLC (Shimadzu, Japan) equipped with a ZAM 4000 evaporative light-scattering detector (ELSD) (Schambeck, Germany). The samples were eluted on a Symmetry C18 column (5 μm, 150 mm × 4.6 mm, Waters) controlled at 30 °C at a flow rate of 1.0 mL/min using methanol/isopropanol (8:2, v/v) as a mobile phase and monitored with the ELSD at 60 °C using nitrogen as a carrier gas at a pressure of 0.5 bar. The conversion of β-sitosterol and the selectivity for β-sitosterol ferulate were defined as follows:
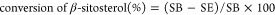

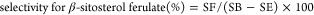
where SB was the molar amount of β-sitosterol at the beginning of the reaction, SE was the molar amount of β-sitosterol at the end of the reaction, and SF was the molar amount of β-sitosterol ferulate at the end of the reaction.

### Structural Characterization

The Fourier transform infrared spectroscopy (FT-IR) spectra of β-sitosterol, ferulic acid, and the purified β-sitosterol ferulate were acquired on a Nicolet IS 50 FT-IR spectrometer (Thermo Fisher Scientific, USA) equipped with a DTGS-KBr detector with a scanning range of 4000–400 cm^–1^, scanning number of 32 times, and resolution of 4.0 cm^–1^. The mass spectrometry (MS) spectrum of β-sitosterol ferulate was acquired by liquid chromatography–ion trap mass spectrometry (Thermo LXQ, Waltham, MA, USA) in positive- and negative ion electrospray ionization (ESI^+^ and ESI^–^) mode with a mass scan range of 50–1000 amu. The other parameters were as described previously.^23^ The nuclear magnetic resonance (NMR) spectrum of the purified product was acquired on an Avance II NMR spectrometer (Bruker, 400 MHz) using CDCl_3_ as the solvent. The ^1^H and ^13^C NMR spectra were obtained at 400 and 100 MHz, respectively.

### Preparation and Purification of Phytosterol Ferulate

By replacing β-sitosterol with phytosterols, we amplified the optimized parameters by a factor of 40 to generate a substantial amount of PF. In brief, 1.96 g of ferulic acid, 1.64 g of phytosterols, 0.196 g of IL, 200 mL of toluene, and a magnetic stirrer were sequentially added to a 500 mL round-bottom flask, and the mixture was reacted at 100 °C for 2.5 h. Upon completion of the reaction, 150 mL of distilled water was added for extraction, and the toluene layer was collected followed by removing the solvent via rotary evaporation, yielding the crude product. In each cycle, 3.0 g of the crude product was separated on a silica gel column (5 × 100 cm) using petroleum ether/ethyl acetate (4:1, v/v) as an eluent to obtain the purified target product PF. The purified PF was analyzed by HPLC-ELSD, and that with a purity of >95% was collected. The PF mainly consisted of 52.1% sitosterol ferulate, 16.8% stigmasterol ferulate, and 26.1% campesterol ferulate, and the average yield of PF after silica gel column chromatography was 55.6%. The above process was repeated several times until 35 g of pure PF was obtained.

### Animals and Diets

Thirty-one specific pathogen-free (SPF) grade male C57BL/6J mice (8 weeks old) were purchased from the Experimental Animal Center of Jiangsu University. After 1 week of adaptation, they were divided into four groups: the low-fat group (LF, *n* = 8), the high-fat group (HF, *n* = 7), the PF group (PF, *n* = 8), and the physical mixture of phytosterols and ferulic acid group (PM, *n* = 8). All mice were housed in SPF animal rooms with a temperature maintained at 25 ± 2 °C, a relative humidity of 50 ± 5%, and a 12 h light/dark cycle. The mice had free access to water and food, and the bedding was changed weekly to maintain a clean and sterile experimental environment. The LF group was fed a basal diet containing only 10% of its energy as fat (Table 1). On the other hand, the remaining three groups were provided with a high-fat diet, where 45% of their energy intake came from fat. Additionally, an extra 0.5% of PF and 0.5% of the mixture of phytosterols and ferulic acid were added to the high-fat diets for the PF and PM groups, respectively (Table 1 and Figure 5). The composition and energy contents of each diet are shown in Table 1.

**Table 1:** Composition of Four Diets in Mice^a^

	**LF**	**HF**	**PF**	**PM**
Ingredient				
casein	200	233.1	233.1	233.1
l-cystine	3	3.5	3.5	3.5
corn starch	452.2	72.8	72.8	72.8
maltodextrin	75	116.5	116.5	116.5
sucrose	172.8	201.4	201.4	201.4
phytosterol ferulate			5	
ferulic acid				1.6
phytosterols				3.5
cellulose	50	58.3	58.3	58.3
soybean oil	25	29.1	29.1	29.1
lard	20	206.8	206.8	206.8
mineral mixture	10	11.6	11.6	11.6
calcium hydrogen phosphate	13	15.2	15.2	15.2
calcium carbonate	5.5	6.4	6.4	6.4
potassium citrate	16.5	19.2	19.2	19.2
vitamin mixture	10	11.6	11.6	11.6
choline bitartrate	2	2.3	2.3	2.3
cholesterol		10	10	10
sodium cholate		2	2	2
FD&C yellow dye #5	0.04		0.05	0.025
FD&C red dye #40	0.01	0.05		0.025
				
total (g)	1055.1	1000.5	1005.5	1005.6
Energy (kcal%)				
protein	20	20.2	20.2	20.2
carbohydrate	70	34.4	34.4	34.4
fat	10	45.4	45.4	45.4
energy density (kcal/g)	3.85	4.68	4.68	4.68

a LF: low-fat group; HF: high-fat group; PF: phytosterol ferulate group; PM: the physical mixture of phytosterols and ferulic acid group.

Throughout the experiment, all mice were provided with fresh food and water daily, and their body weight and food intake were recorded weekly. The feeding experiment was conducted for 15 weeks. The feces were collected during the final week. At the end of week 15, the mice were fasted for 12 h, then anesthetized with isoflurane, followed by blood sampling from their orbital veins, and then sacrificed. Their livers, kidneys, hearts, testicles, perirenal fat, and epididymal fat were collected, weighed, and preserved at −80 °C until analysis. All animal experiment operations were approved by the Animal Experiment Ethics Committee of Jiangsu University (UJS-IACUC-2020070202).

### Measurement of Plasma Lipids

Freshly drawn blood was immediately placed in centrifuge tubes containing heparin sodium and then centrifuged at 3000 rpm for 10 min. The obtained plasma was then stored at −80 °C until analysis. The determination of plasma total cholesterol (TC), triacylglycerols (TG), and high-density lipoprotein cholesterol (HDL-C) was carried out according to the instructions provided in the corresponding commercial reagent kit (Nanjing Jiancheng Bioengineering Institute, Nanjing, China). Non-HDL-C, referring to the amount of low-density lipoprotein cholesterol and very-low-density lipoprotein cholesterol, was calculated by subtracting HDL-C from TC.

### Histologic Examination of the Liver

First, small pieces of fresh liver tissues were fixed in 10 mL of a 10% formalin solution followed by alcohol dehydration and xylene treatment. Subsequently, the transparent liver tissue was embedded in paraffin and then cut into 5 μm-thick sections. These sections were treated with xylene deparaffinization, stained with hematoxylin-eosin (HE), and preserved with neutral gel. Finally, the morphology of the liver tissue was observed under a BX53-P microscope (Olympus, Tokyo, Japan).

### Measurement of Hepatic and Fecal Lipids

The extraction of hepatic and fecal lipids was carried out according to the method described by He et al.^2^ One of the above hepatic or fecal extracts was added to 100 μL of boron trifluoride methylation solution containing 6 mg/mL heptadecanoic acid (Sigma-Aldrich, Shanghai, China) as an internal standard (IS) to produce the corresponding fatty acid methyl esters. These methyl esters were then analyzed and quantified using gas chromatography. The specific instruments and operation parameters were the same as in our previous study.^24,25^ The content of each fatty acid in the liver and feces was calculated according to the amount of IS added before methylation.

### Measurement of Hepatic and Fecal Sterols

The liver cholesterol was measured as the method described in our previous study.^2^ In brief, the above-mentioned liver lipid extract was added with 200 μL of 6 mg/mL phytostanols as an IS and 1 mL of 2 mol/L sodium hydroxide in absolute ethanol and saponified at 70 °C for 2 h. The saponified matter was extracted with chloroform, dried with nitrogen, and redissolved in anhydrous ethanol for HPLC analysis. The cholesterol content in liver extracts was analyzed by the same method as in the previous section of HPLC analysis. Similarly, the above-mentioned fecal lipid extract was saponified with ergosterol as an IS. The method for analyzing fecal cholesterol was consistent with the previously described method, except for the mobile phase composition, which consisted of acetonitrile/methanol (7:3, v/v). Each sterol in the liver and feces was calculated according to the amount of its respective IS added.

### Statistical Analyses

The results were expressed as means ± standard deviations. The SPSS Statistics 20 software (SPSS Inc.) was used for one-way analysis of variance (ANOVA) followed by the post hoc LSD test. The values marked with different letters were significantly different (*p* < 0.05).

## Results

### TLC and HPLC Analyses

**Figure 1:**
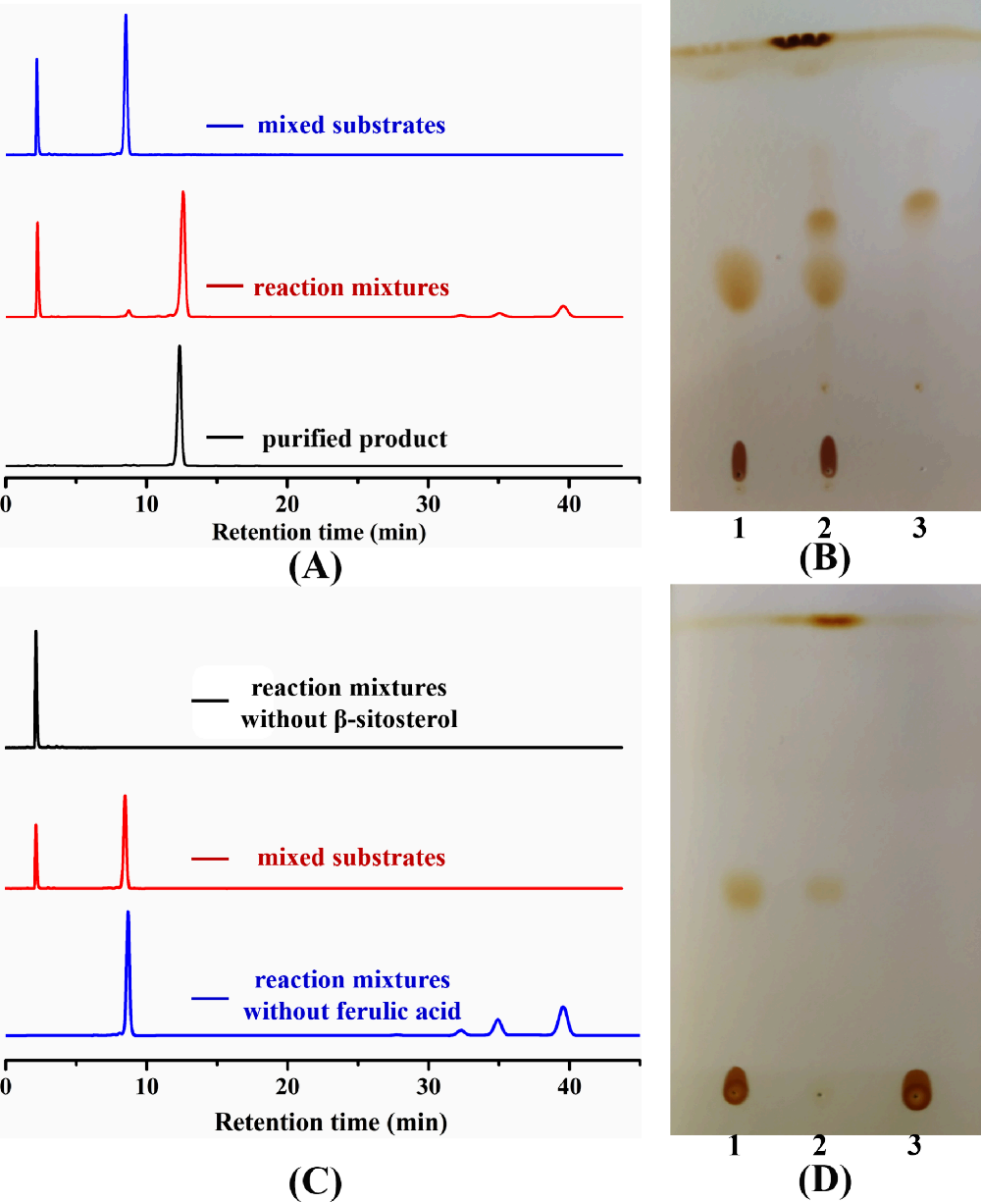
(A) HPLC chromatograms of mixed substrates (upper curve), reaction mixtures (middle curve), and the purified product (lower curve); (B) TLC chromatograms of mixed substrates (left, sample 1), reaction mixtures (middle, sample 2), and the purified product (right, sample 3); (C) HPLC chromatograms of reaction mixtures in the absence of β-sitosterol (upper curve) or ferulic acid (lower curve) and mixed substrates (middle curve); (D) TLC chromatograms of reaction mixtures in the absence of β-sitosterol (right, sample 3) or ferulic acid (middle, sample 2) and mixed substrates (left, sample 1).

As shown in Figure 1A,B, the *R*_f_ values of β-sitosterol and ferulic acid were 0.41–0.43 and 0–0.07, respectively, while that of β-sitosterol ferulate was 0.56–0.58. Correspondingly, the two substrates, β-sitosterol and ferulic acid, were eluted at retention times of 8.5 and 2.2 min, respectively, and the retention time of β-sitosterol ferulate was 12.7 min.

### FT-IR Analysis

**Figure 2:**
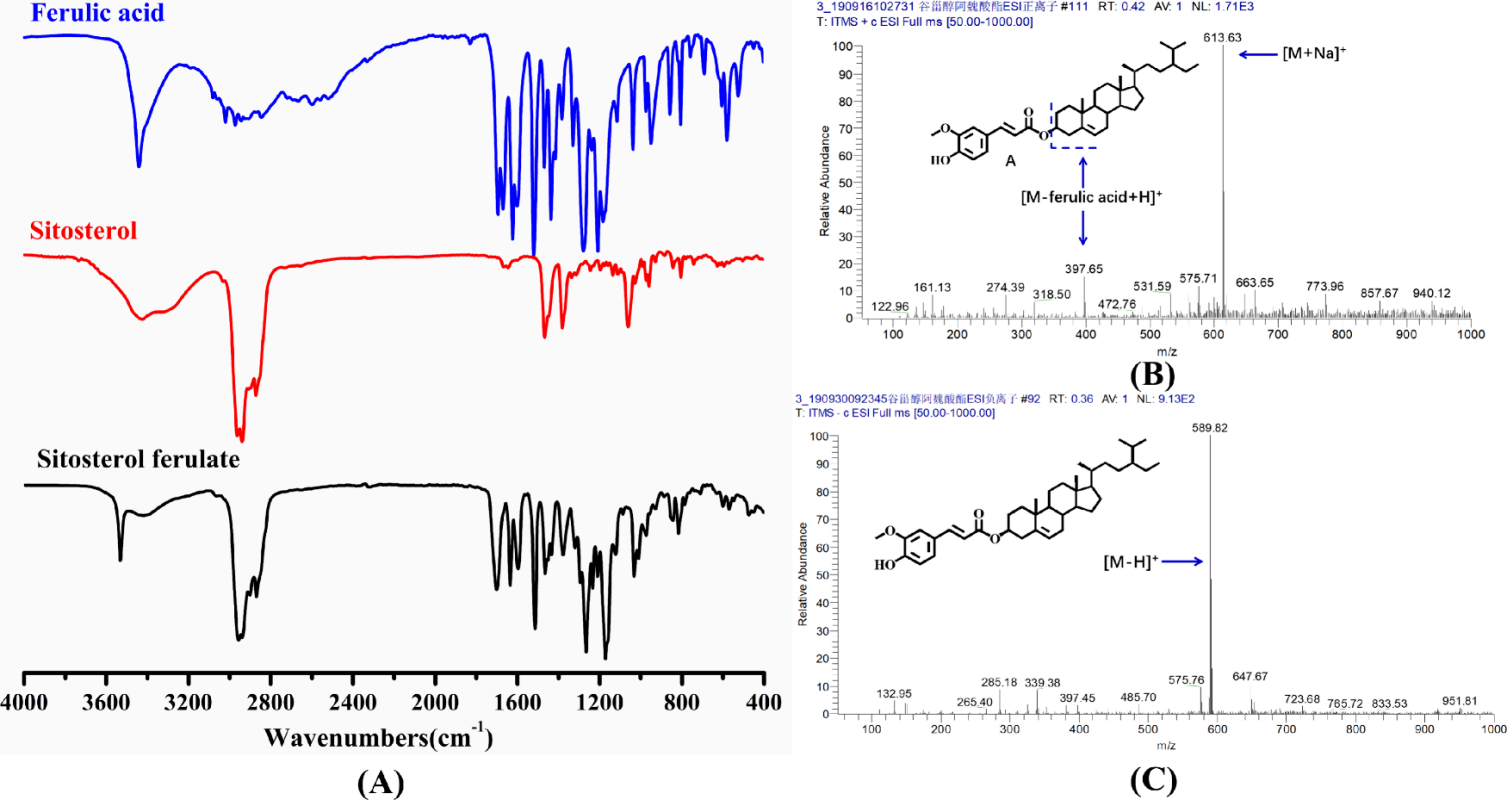
(A) FT-IR spectra of ferulic acid (upper curve), β-sitosterol (middle curve), and the product (lower curve); (B) mass spectrum of the product in ESI positive ion mode; (C) mass spectrum of the product in ESI negative ion mode.

The FT-IR spectra of ferulic acid, β-sitosterol, and their esterified products are shown in Figure 2A. The upper curve (ferulic acid) showed three characteristic absorption signals. The sharp peak at 3438 cm^–1^ was attributed to the stretching vibration of the hydroxyl groups. The broad peaks between 2100 and 3100 cm^–1^ corresponded to the characteristic absorption of carboxyl groups. The peak at 1690 cm^–1^ was the stretching vibration of C=O in the carboxyl groups. In the middle curve, β-sitosterol exhibited a characteristic absorption signal of hydroxyl groups at 3423 cm^–1^ with moderate intensity. In the lower curve, the product showed three characteristic absorption peaks. The peak at 3423 cm^–1^ corresponded to hydroxyl groups. The peaks at 1701 and 1172 cm^–1^ were characteristics of C=O and C–O in the ester bond, respectively. Compared with the FT-IR spectra of ferulic acid and β-sitosterol, the absorption signal of carboxyl groups in the product disappeared while the characteristic peaks of C=O and C–O in the ester bond appeared, indicating that the product is the target β-sitosterol ferulate.

### MS Analysis

In the ESI^+^ mode, the molecular ion peaks of the compounds often showed [M + Na]^+^ or [M + H]^+^ signals.^18^ In the ESI^–^ mode, [M–H]^−^ or [M + Cl]^−^ signals were often present. The relative molecular masses of β-sitosterol and ferulic acid were *m*/*z* 414 and *m*/*z* 194, respectively, and the relative molecular mass of their esterified product, β-sitosterol ferulate, was *m*/*z* 590. As shown in Figure 2B, the peak at *m*/*z* 613 corresponded to the [M + Na]^+^ ion of β-sitosterol ferulate in the ESI^+^ mode. Meanwhile, the peak at *m*/*z* 397 corresponded to the [M-ferulic acid + H]^+^ ion, which was the major fragment of β-sitosterol ferulate. In Figure 2C, the base peak at *m*/*z* 589 was observed in the ESI^–^ mode, which corresponded to the [M–H]^−^ ion of β-sitosterol ferulate. The MS results provided further support for the successful synthesis of β-sitosterol ferulate.

### NMR Analysis

**Figure 3:**
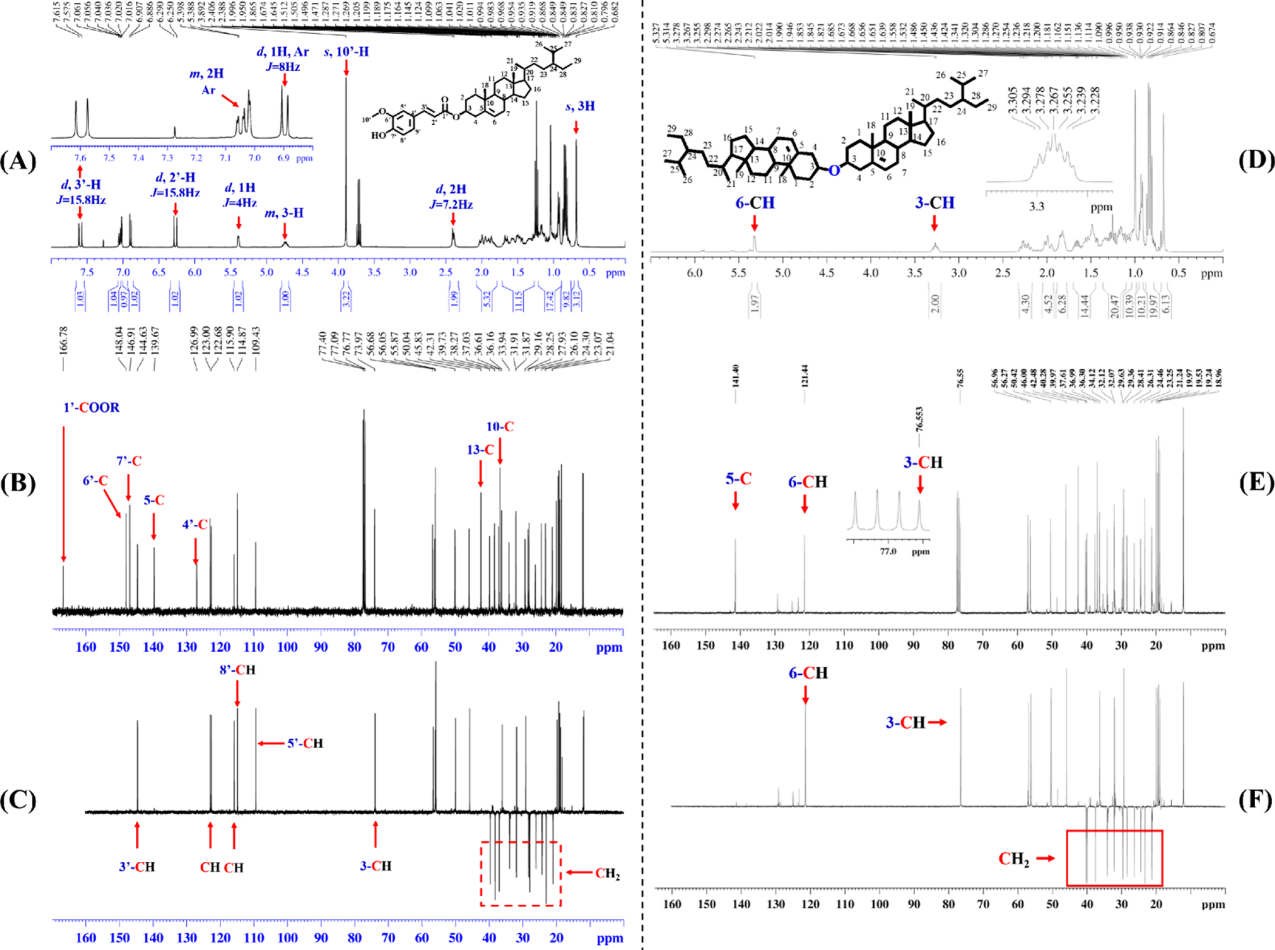
(A) Chemical structure of the target product β-sitosterol ferulate and ^1^H NMR spectrum of the product; (B) ^13^C NMR spectrum of the product; (C) DEPT-135 NMR spectrum of the product; (D) chemical structure of the byproduct disitosterol ether and its ^1^H NMR spectrum; (E) ^13^C NMR spectrum of the byproduct; (F) DEPT-135 spectrum of the byproduct.

The product isolated by silica gel column chromatography (purity >98%) was subjected to NMR analysis. Figure 3 shows the chemical structure of β-sitosterol ferulate and the ^1^H, ^13^C, and DEPT-135 NMR spectra of the product. The chemical shifts of the ^1^H and ^13^C spectra of the synthesized product were assigned by comparison to the NMR data of β-sitosterol (not shown).

In Figure 3A, the signals of hydrogen protons in the sterol skeleton mainly appeared below 4.8 ppm, whereas those in the ferulic acid skeleton mainly appeared above 4.8 ppm, consistent with the spectrum reported by Condo et al.^12^ Due to the complex skeleton of β-sitosterol, the signals of hydrogen protons on the methylene group overlapped in the chemical shift range between 1.1 and 1.6 ppm. The peaks below 1.1 ppm were mainly ascribed to the resonance of hydrogen protons on C*H*_3_, and those between 1.6 and 3.5 ppm mainly corresponded to C*H*. In β-sitosterol, 3-H in C*H* appeared as a multiplet at a chemical shift of 3.5 ppm, while in the product, it shifted to 4.7 ppm. This was consistent with an earlier study in which the resonance of 3-H in C*H* was reported at 4.75 ppm in the ^1^H NMR spectrum of stigmasterol *trans*-ferulate.^26^ These results suggested the existence of an ester bond in the product. The other peaks at 7.6, 7.0, 6.9, 6.2, 5.4, and 3.9 ppm were the resonance signals of the hydrogen protons on the ferulic acid skeleton. Of them, the singlet at a chemical shift of 3.9 ppm was the resonance signal of hydrogen in 10′-OC*H*_3_. However, the 7′-H resonance signal of the phenolic hydroxyl group was not found in the ^1^H NMR spectrum because an active hydrogen has no fixed chemical shift.

In general, all carbon atoms of the test compound appear in a ^13^C NMR spectrum.^27^ In Figure 3B, the resonance absorption signals of 39 carbon atoms were observed in the ^13^C NMR spectrum. In Figure 3C, no quaternary carbon (*C*) appears and only the resonance signals of 32 carbon atoms can be seen in the DEPT-135 spectrum. The seven missing signals were those at 166.7, 148.0, 146.9, 139.6, 136.9, 42.3, and 36.6 ppm in the ^13^C NMR spectrum, which correspond to quaternary carbons: 1′-*C*, 6′-*C*, 7′-*C*, 5-*C*, 4′-*C*, 13-*C*, and 10-*C*, respectively. The other carbons showed various peak shapes. Specifically, primary (−*C*H_3_) and tertiary (−*C*H) carbons showed positive peaks, whereas secondary carbons (−*C*H_2_) showed an inverted peak.^27^ These results were highly consistent with the chemical structure of β-sitosterol ferulate. Furthermore, the downward peaks between 20 and 40 ppm corresponded precisely to the −*C*H_2_ group of the product. The upward peaks below 20 and above 29 ppm were mainly ascribed to −*C*H_3_ and −*C*H, respectively, of the product. These results agreed with those of Condo et al.^12^ Taken together, the TLC, HPLC, FT-IR, MS, and NMR analyses conclusively demonstrated that the product was the target. Therefore, β-sitosterol ferulate was successfully synthesized by using acidic IL as a catalyst.

### Identification of the Byproduct

Some unknown products appeared in the reaction mixture, corresponding to peaks with retention times of 32–39 min in Figure 1A and spots with *R*_f_ values of 0.98–1.00 in Figure 1B. This indicated that side reactions occurred during the IL-catalyzed esterification of β-sitosterol with ferulic acid. To identify the byproducts, the reaction was repeated in the absence of β-sitosterol or ferulic acid, and the results are shown in Figure 1C,D. In the absence of ferulic acid, we observed distinct byproduct spots with *R*_f_ values of 0.98–1.00, corresponding to peaks with retention times of 32–39 min. In the absence of β-sitosterol, no byproduct was observed. Therefore, the byproducts were derived from β-sitosterol itself in the presence of acidic ILs.

The most abundant byproduct was purified by silica gel column chromatography and then used for NMR analyses, as shown in Figure 3D–F. Previous studies have shown that sterols may undergo intramolecular dehydration or oxidation reactions under high temperature conditions.^28,29^ However, the number of H atoms in the ^1^H spectrum of the byproduct was almost twice that of β-sitosterol (Figure 3D). The results from TLC and HPLC analyses (Figure 1C,D) showed that the byproducts were highly hydrophobic. These results suggested that two molecules of β-sitosterol possibly underwent etherification in the presence of acidic ILs. The 3-H and 3-C chemical shifts of β-sitosterol were 3.52 and 71.90 ppm, but they were shifted to 3.26 and 76.55 ppm in the byproducts (Figure 3D–F), respectively, which meant that the alcoholic hydroxyl group in C-3 disappeared. According to the above analysis, the byproduct was disitosterol ether, the etherification product of two molecules of β-sitosterol. Consistently, Kaufmann et al. also noted dehydration under formation of an ether linkage between two sterols.^30^ The toxicity of disitosterol ether was currently unknown, and β-sitosterol ferulate containing disitosterol ether should not be incorporated directly into food unless the disitosterol ether was completely removed to meet the food requirements.

### Optimization of Reaction Parameters

In addition to β-sitosterol ferulate, some unknown byproducts were also simultaneously produced in the IL-catalyzed esterification of β-sitosterol with ferulic acid. Therefore, process optimization was required to increase the yield of β-sitosterol ferulate and reduce the formation of byproducts. To this end, the effects of different ILs, IL dose, reaction temperature, substrate molar ratio, substrate concentration, and reaction time on the conversion of β-sitosterol and the selectivity for the product were investigated to determine the optimal reaction parameters.

#### IL Screening

In this study, we investigated the catalytic efficacy of the three acidic ILs including [BSO_3_HMim]HSO_4_, [BSO_3_HMim]TS, and [BSO_3_HMim]OTF in the esterification of β-sitosterol with ferulic acid. The use of [BSO_3_HMim]HSO_4_ and [BSO_3_HMim]TS failed to catalyze the formation of β-sitosterol ferulate, and only [BSO_3_HMim]OTF was successful. The conversion of β-sitosterol was 51.1%, and the selectivity for the product was 75.5%, when [BSO_3_HMim]OTF was used as a catalyst. The three ILs described above have the same cation ([BSO_3_HMim]^+^) but different anions ([HSO_4_]^−^, [TS]^−^, and [OTF]^−^). Therefore, we speculated that the anions of the ILs played a key role in this esterification process. We then investigated the effect of acidic ILs with different cations and the same anion, [OTF]^−^, on the conversion of β-sitosterol. These ILs included 1-butyl-3-methylimidazolium trifluoromethanesulfonate ([BMIM]OTF), tetramethylguanidine trifluoromethanesulfonate, *N*-butyl, methylpyrrolidinium trifluoroacetate, and *N*-butylsulfonate pyridinium trifluoromethanesulfonate ([BSO_3_Py]OTF). Of these ILs, only [BSO_3_Py]OTF successfully catalyzed the synthesis of β-sitosterol ferulate, indicating that not all ILs with the [OTF]^−^ anion can catalyze the esterification of β-sitosterol and ferulic acid. Notably, [BSO_3_HMim]OTF can catalyze esterification, whereas [BMIM]OTF cannot. The only difference between the two was that the cation of the former was sulfonated. This implied that ILs capable of catalyzing this esterification should combine anion [OTF]^−^ with a cation containing a sulfonic acid group. In this study, the conversion rate of β-sitosterol catalyzed by [BSO_3_Py]OTF was lower than that catalyzed by [BSO_3_HMim]OTF. Therefore, [BSO_3_HMim]OTF was selected as the catalyst for the following experiments.

#### IL Dose

**Figure 4:**
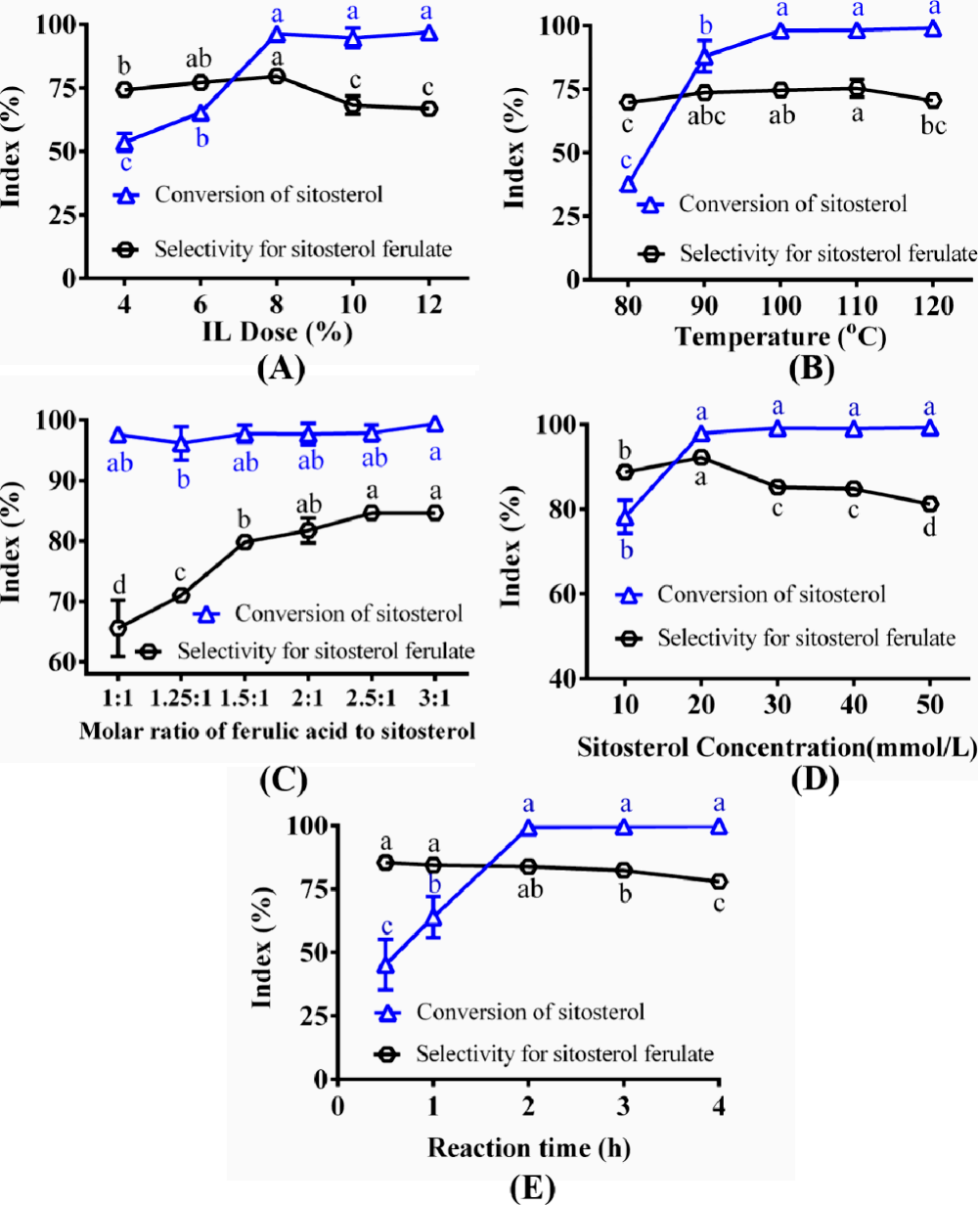
Effect of reaction parameters on the conversion of β-sitosterol and the selectivity of β-sitosterol ferulate. (A) IL dose: 5 mL of toluene, [BSO_3_HMIM]OTF, 30 mmol/L β-sitosterol, 1.5:1 molar ratio of ferulic acid to β-sitosterol, 100 °C, 2 h; (B) reaction temperature: 5 mL of toluene, 8% [BSO_3_HMIM]OTF (w/w, relative to the total amount of both substrates), 30 mmol/L β-sitosterol, 1.5:1 molar ratio of ferulic acid to β-sitosterol, 2 h; (C) molar ratio of ferulic acid to β-sitosterol: 5 mL of toluene, 8% [BSO_3_HMIM]OTF (w/w, relative to the total amount of both substrates), 30 mmol/L β-sitosterol, 100 °C, 2 h; (D) β-sitosterol concentration: 5 mL of toluene, 8% [BSO_3_HMIM]OTF (w/w, relative to the total amount of both substrates), 2.5:1 molar ratio of ferulic acid to β-sitosterol, 100 °C, 2 h; (E) reaction time: 5 mL of toluene, 8% [BSO_3_HMIM]OTF (w/w, relative to the total amount of both substrates), 20 mmol/L β-sitosterol, 2.5:1 molar ratio of ferulic acid to β-sitosterol, 100 °C.

The effect of the [BSO_3_HMim]OTF dose on the conversion of β-sitosterol and the selectivity for the product was investigated by varying the IL dose from 4 and 12% (Figure 4A). Almost no product was synthesized in the absence of [BSO_3_HMim]OTF (data not shown). The conversion of β-sitosterol reached 53.6%, and the selectivity for β-sitosterol ferulate reached 74.3% when using 4% IL as a catalyst. With an increasing dose of the IL, the conversion rate of β-sitosterol increased rapidly, while the product selectivity was almost unchanged. With an IL dose of 8%, the conversion rate of β-sitosterol was 96.3% and the product selectivity was 79.6%. Under these conditions, the yield of β-sitosterol ferulate reached 76.7%. When the amount of [BSO_3_HMim]OTF exceeded 8%, a further increase in the dose had no significant effect on the conversion rate of β-sitosterol, which remained above 95%, but the product selectivity gradually decreased, indicating that 8% [BSO_3_HMim]OTF was sufficient for this reaction. Therefore, 8% [BSO_3_HMim]OTF was used for the subsequent experiments.

#### Reaction Temperature

The reaction temperature was an important factor for esterification reactions due to its effects on the substrate solubility in the solvent and the molecular collision rate. The influence of reaction temperature on the conversion of β-sitosterol and the selectivity for the product was investigated from 80 to 120 °C. In Figure 4B, as the reaction temperature increased from 80 to 100 °C, the conversion of β-sitosterol first increased rapidly and then slowly and reached a maximum of 98.1% at 100 °C. This can be ascribed to the promotion of mass transfer, and thus conversion, at higher temperature.^31^ Above 100 °C, the conversion rate of β-sitosterol remained unchanged with a further increase in temperature. Meanwhile, from 80 to 110 °C, the selectivity for β-sitosterol ferulate was essentially constant, but it decreased at temperatures above 110 °C, suggesting that side reactions were more serious at excessive temperature. Both the conversion of β-sitosterol and the product selectivity reached their maxima at 100 and 110 °C, respectively, with no significant difference in conversion or selectivity between the two temperatures. To minimize economic cost, a reaction temperature of 100 °C was selected for the subsequent experiments.

#### Influence of the Molar Ratio of Ferulic Acid to β-Sitosterol

The influence of the molar ratio of ferulic acid to β-sitosterol, in the range of 1:1–3:1, on the conversion of β-sitosterol and the selectivity for β-sitosterol ferulate was investigated. In Figure 4C, the conversion rate of β-sitosterol exceeded 95% throughout the investigated molar ratio range. However, the selectivity for β-sitosterol ferulate exhibited a more complex trend. Specifically, at an equimolar ratio of ferulic acid to β-sitosterol, the selectivity was approximately 65.5%. Shifting the reaction equilibrium toward esterification proved to be difficult with an equimolar substrate ratio. The high conversion rate of β-sitosterol combined with the low selectivity for β-sitosterol ferulate can be attributed to the greater prevalence of side reactions at an equimolar substrate ratio. However, as a reversible reaction, increasing the substrate molar ratio pushed the reaction equilibrium toward ester generation, thereby increasing the amount of target ester. When the molar ratio was increased from 1:1 to 1.5:1, the selectivity for β-sitosterol ferulate was significantly improved, reaching 79.8%. The selectivity continued to gradually increase as the molar ratio was raised further, reaching a maximum of 84.7% at a ratio of 2.5:1, corresponding to a β-sitosterol ferulate yield of 82.9%. Therefore, the molar ratio of ferulic acid to β-sitosterol was selected as 2.5:1 for the subsequent experiments.

#### Influence of the β-Sitosterol Concentration

The β-sitosterol concentration range of 10–50 mmol/L was used to study the corresponding effect on the conversion rate of β-sitosterol and the selectivity for β-sitosterol ferulate (Figure 4D). At 10 mmol/L, the conversion of β-sitosterol reached 78.2%. The conversion of β-sitosterol showed a linear increase with the concentration, reaching 97.9% at a β-sitosterol concentration of 20 mmol/L. Between the β-sitosterol concentrations of 10 and 20 mmol/L, the selectivity for β-sitosterol ferulate was approximately constant. As the concentration of β-sitosterol was further increased from 20 to 50 mmol/L, the conversion rate of β-sitosterol remained almost unchanged, but the selectivity for β-sitosterol ferulate gradually decreased. Therefore, the concentration of β-sitosterol ferulate approached the maximum at a β-sitosterol concentration of 20 mmol/L.

#### Reaction Time

The effect of the reaction time on the conversion of β-sitosterol and the selectivity for β-sitosterol ferulate was investigated under the selected parameters in the range of 0.5–4 h. In Figure 4E, the conversion of β-sitosterol after 0.5 h was 45.2%. Prolonging the reaction time significantly increased the conversion rate of β-sitosterol, reaching a maximum value of 99.4% after 2 h of reaction. Within the 0.5–2 h reaction period range, the selectivity for the product did not change significantly. Further extension of the reaction time did not increase the conversion rate of β-sitosterol but did reduce the selectivity. This indicated that the esterification of β-sitosterol with ferulic acid had approximately reached equilibrium at 2 h, and an extension of the reaction time merely increased the formation of byproducts. Therefore, 2 h was sufficient for esterification to reach equilibrium.

### Body Weight, Food Intake, and Relative Organ Weights

The body weight, food intake, and relative organ weight of each group of mice are shown in Table 2. The initial body weights of the four groups of mice were similar. Although the final body weight of the HF mice did not increase significantly compared with LF, their weight gain was significantly increased. PF supplementation caused a 20.8% (*p* > 0.05) reduction in weight increment. Mice fed a high-fat diet containing HF, PF, and PM had a lower food intake than mice fed a low-fat diet, which was attributed to the lower energy density of the LF diet. Compared to LF, the other three groups experienced a significant reduction in relative heart and kidney weights. However, PF and PM did not alter these two indices.

**Table 2:** Body Weight, Food Intake, and Relative Organ Weights of Mice in Each Group^a^

	**LF**	**HF**	**PF**	**PM**	*P* value
Body weight (g)					
initial	24.0 ± 1.5^a^	23.9 ± 1.0^a^	24.1 ± 1.1^a^	24.0 ± 1.3^a^	0.99
final	28.4 ± 2.3^b^	30.3 ± 2.2^ab^	29.4 ± 1.7^ab^	31.2 ± 3.0^a^	0.08
weight gain	4.4 ± 1.1^c^	6.7 ± 1.9^ab^	5.3 ± 1.5^bc^	7.2 ± 2.7^a^	0.01
daily food intake (g per mouse)	2.7 ± 0.3^a^	2.1 ± 0.3^c^	2.5 ± 0.3^ab^	2.4 ± 0.3^b^	0.00
Relative organ weights (%)					
liver	3.29 ± 0.2	3.26 ± 0.2	3.37 ± 0.2	3.34 ± 0.2	0.64
heart	0.54 ± 0.07^a^	0.43 ± 0.03^b^	0.44 ± 0.05^b^	0.46 ± 0.08^b^	0.00
kidney	1.13 ± 0.08^a^	0.97 ± 0.03^b^	1.01 ± 0.16^b^	0.96 ± 0.06^b^	0.02
testes	0.64 ± 0.14	0.63 ± 0.09	0.64 ± 0.07	0.62 ± 0.13	0.88
perirenal fat	0.51 ± 0.22^b^	1.15 ± 0.17^a^	0.61 ± 0.29^b^	1.10 ± 0.49^a^	0.00
epididymis fat	2.04 ± 0.59^b^	3.98 ± 0.91^a^	2.50 ± 0.93^b^	3.67 ± 1.38^a^	0.00

a The data marked with different superscript letters represents significant differences in the same row (*p* < 0.05). LF: low-fat group; HF: high-fat group; PF: phytosterol ferulate group; PM: the physical mixture of phytosterols and ferulic acid group.

High-fat feeding led to significant increases in relative kidney and epididymal fat weight in mice (HF vs LF), which were reversed by the addition of PF. Compared to HF, PF caused a reduction of perirenal fat and epididymal fat by 46.9% (*p* < 0.05) and 37.1% (*p* < 0.05), respectively. However, PM showed no significant difference with HF in terms of perirenal and epididymal fat.

### Plasma TC, TG, HDL-C, non-HDL-C, and HDL-C/TC

**Figure 5:**
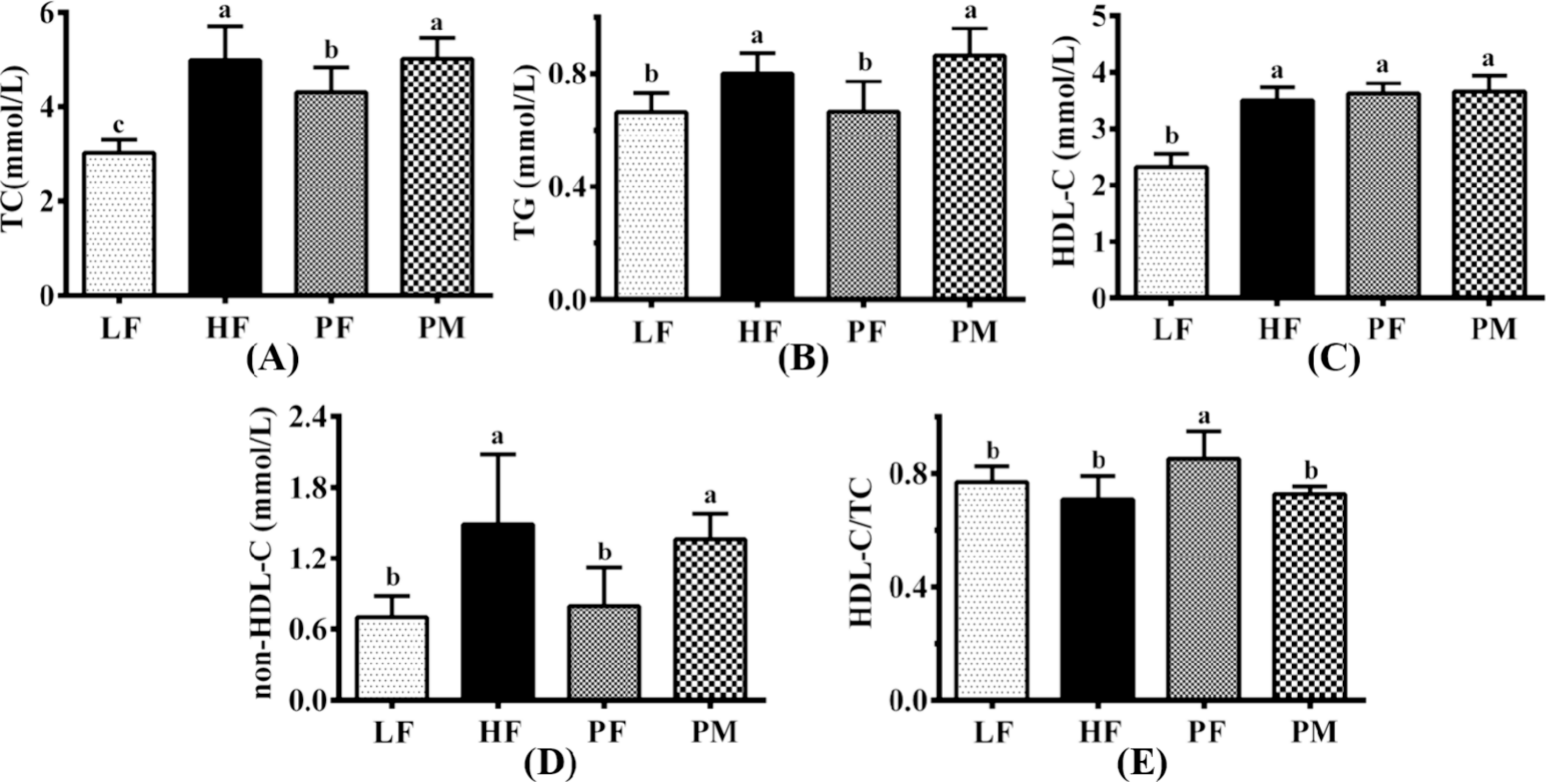
Plasma total cholesterol (TC, A), triglyceride (TG, B), high-density lipoprotein cholesterol (HDL-C, C), non-HDL-C (D), and HDL-C/TC (E) in four groups of mice. LF: low-fat group (*n* = 8); HF: high-fat group (*n* = 7); PF: phytosterol ferulate group (*n* = 8); PM: physical mixture of phytosterols and ferulic acid group (*n* = 8). Data are expressed as the means with different superscript letters (a, b, and c) that differ significantly at *p* < 0.05.

The plasma TG, TC, HDL-C, non-HDL-C, and HDL-C/TC ratios in the mice of each group are shown in Figure 5. Compared to LF, the levels of plasma TC, TG, HDL-C, and non-HDL-C in the HF mice increased significantly by 39.3, 20.7, 50.5, and 112.9%, respectively, indicating the successful establishment of a high-fat model. Compared to HF, plasma TC, TG, and non-HDL-C of PF mice decreased by 13.7, 16.9, and 46.3%, respectively. PF supplementation significantly increased the plasma HDL-C/TC ratio but had no effect on plasma HDL-C. In contrast, PM did not cause significant changes in these plasma lipid levels.

### Liver Fatty Acids

**Figure 6:**
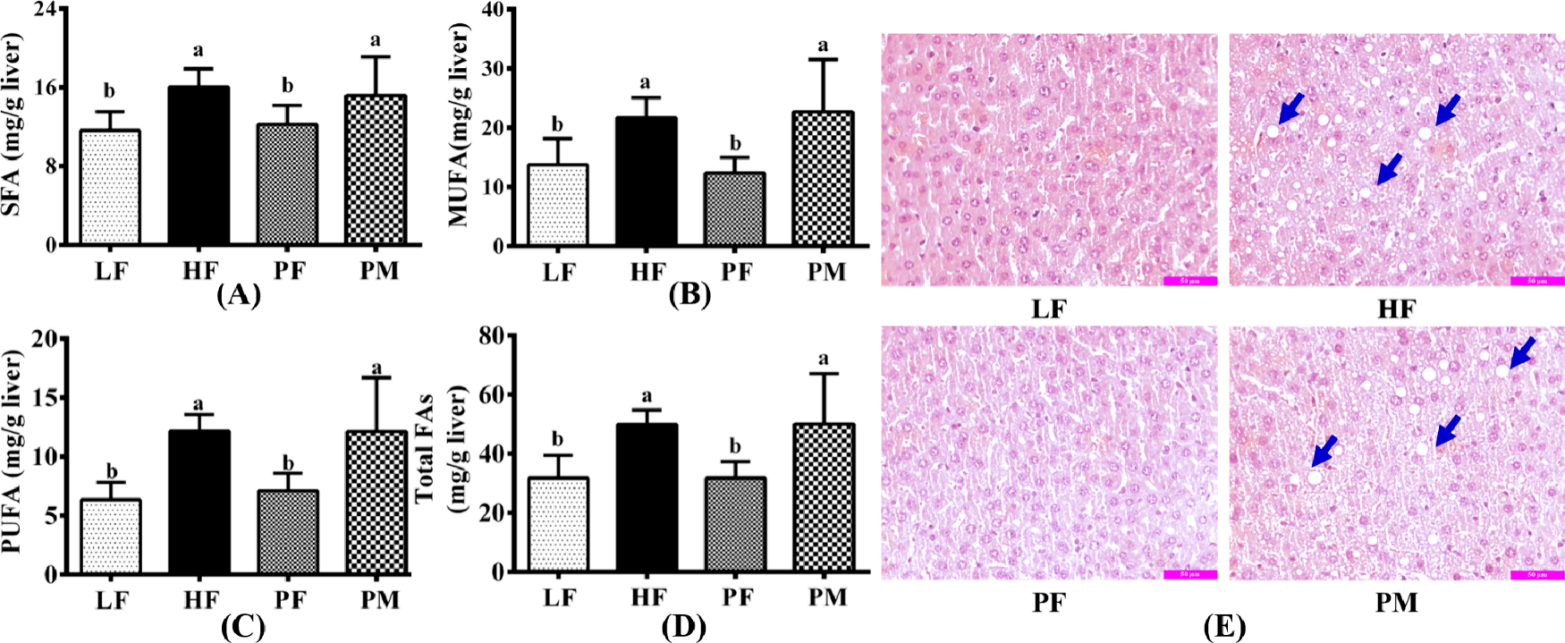
Liver saturated fatty acid (SFA, A), monounsaturated fatty acid (MUFA, B), polyunsaturated fatty acid (PUFA, C), and total fatty acid (total FA, D) contents in four groups of mice. HE stained section of the liver tissue (E). LF: low-fat group (*n* = 8); HF: high-fat group (*n* = 7); PF: phytosterol ferulate group (*n* = 8); PM: physical mixture of phytosterols and ferulic acid group (*n* = 8). Data are expressed as the means with different superscript letters (a and b) that differ significantly at *p* < 0.05.

Fatty acids, the hydrolytic products of triglycerides, serve as indicators of lipid levels. The total fatty acids (FAs) in the liver can be categorized into saturated fatty acids (SFAs, e.g., palmitic acid and stearic acid), monounsaturated fatty acids (MUFAs, e.g., oleic acid and palmitoleic acid), and polyunsaturated fatty acids (PUFAs, e.g., linoleic acid and linolenic acid). The content of fatty acids in the livers of mice from each group is depicted in Figure 6A–D. A comparison between the LF and HF groups revealed that the levels of SFAs, MUFAs, PUFAs, and total FAs in the livers of HF mice increased by 27.5, 36.4, 48.3, and 36.5%, respectively (*p* < 0.05), indicating that a long-term high-fat diet led to significant fat accumulation in the liver, increasing the risk of fatty liver formation. In contrast, the PF group demonstrated significantly reduced levels of SFAs, MUFAs, PUFAs, and total FAs in the liver, with decreases of 23.1, 42.8, 41.8, and 36.4%, respectively (*p* < 0.05), which were similar to those in the LF group. These findings suggest that PF can effectively reduce the liver fatty acid content and inhibit lipid accumulation in the liver. No significant difference was observed in liver lipid levels between the PM and HF groups. However, a comparison between PM and PF groups revealed significantly reduced levels of all types of fatty acids in the liver of the PF group, with SFAs, MUFAs, PUFAs, and total FAs decreasing by 19.0, 45.3, 41.2, and 35.1%, respectively. This indicates that PF exhibited a significantly superior inhibitory effect on liver lipid aggregation compared to PM at the same dosage.

### Liver Histological Observation

Figure 6E displays the morphologies of liver tissue sections from mice in each group following HE staining. In the LF group, the liver cells exhibited normal morphology with no visible lipid droplets. The HF group, however, demonstrated swollen liver cells that were loosely arranged and disordered, containing numerous large lipid droplets (blue arrow). This suggests that the prolonged intake of a high-fat diet led to the accumulation of fat in the liver. There were no significant disparities in the number of lipid droplets between the PM and HF groups (blue arrow). The PF group showcased normal liver cell morphology, similar to that of the LF group, with no apparent lipid droplets. The results were highly consistent with previously obtained data regarding relative fat weights, plasma TG, and hepatic fatty acids, suggesting that PF effectively inhibited fatty accumulation caused by long-term high-fat diet consumption.

### Liver Cholesterol

**Figure 7:**
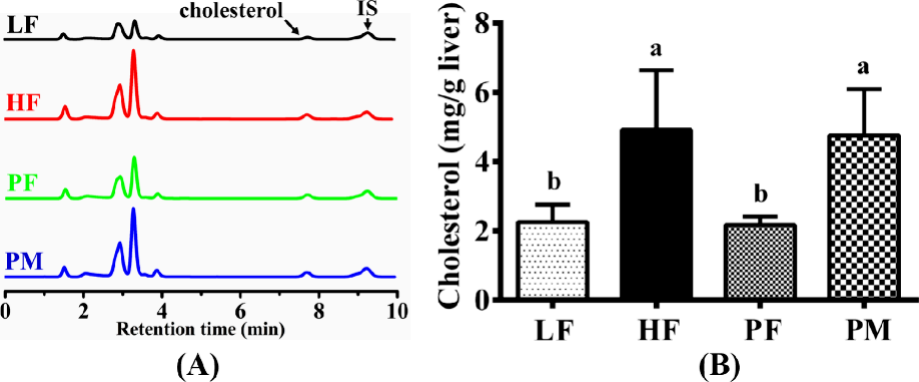
High-performance liquid chromatograms of liver cholesterol (A) and its content (B) in four groups of mice. LF: low-fat group (*n* = 8); HF: high-fat group (*n* = 7); PF: phytosterol ferulate group (*n* = 8); PM: physical mixture of phytosterols and ferulic acid group (*n* = 8); IS: internal standard. Data are expressed as the means with different superscript letters (a and b) that differ significantly at *p* < 0.05.

The liver serves as the primary site for cholesterol synthesis and metabolism, and its cholesterol content is a crucial indicator for evaluating liver lipid levels. Figure 7A displays the liquid chromatography profiles of sterols in the liver of mice in each group. Obviously, cholesterol and IS (phytostanols) were eluted at 7.8 and 9.2 min, respectively. The cholesterol content in the livers of mice from each group is presented in Figure 7B. The HF group exhibited a 117% increase compared to that of the LF group. PF supplementation led to a 55.4% reduction in the liver cholesterol content, whereas PM showed no significant influence. The PF group demonstrated a 54.3% reduction compared to the PM group, highlighting the greater effectiveness of PF in reducing the liver cholesterol content.

### Fecal Fatty Acids

**Figure 8:**
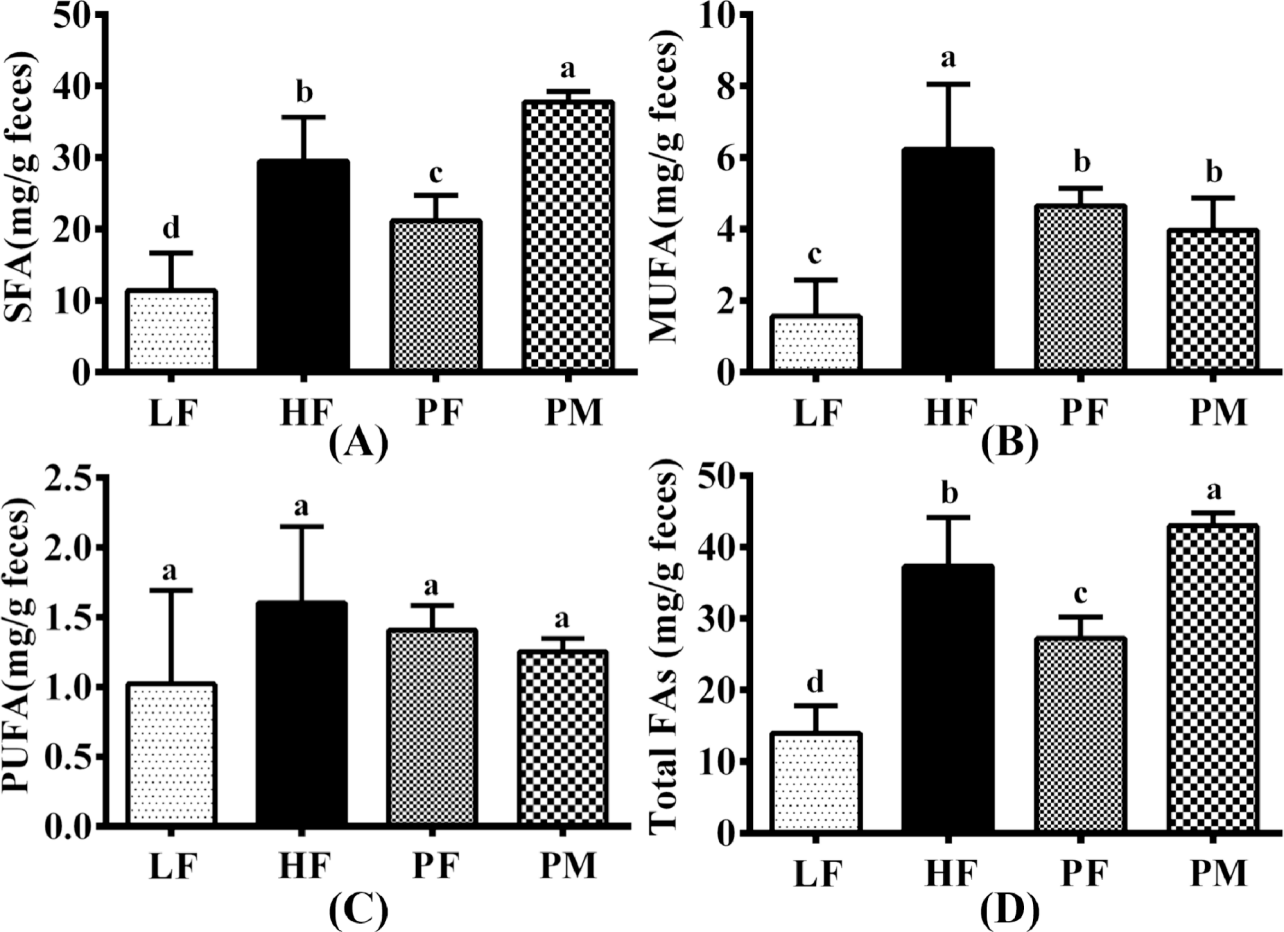
Fecal saturated fatty acid (SFA, A), monounsaturated fatty acid (MUFA, B), polyunsaturated fatty acid (PUFA, C), and total fatty acid (total FA, D) contents in four groups of mice. LF: low-fat group (*n* = 8); HF: high-fat group (*n* = 7); PF: phytosterol ferulate group (*n* = 8); PM: physical mixture of phytosterols and ferulic acid group (*n* = 8). Data are expressed as the means with different superscript letters (a, b, c, and d) that differ significantly at *p* < 0.05.

Fatty acids in feces can provide some insight into the degree of lipid absorption in the small intestine. As illustrated in Figure 8, the composition of fatty acids in the feces of mice in each group consisted primarily of SFAs followed by MUFAs and the least PUFAs. When compared to the LF group, the content of various fatty acids in the feces of mice in the HF group exhibited a significant increase. In particular, the levels of SFAs, MUFAs, PUFAs, and total FAs have risen by 49.1, 74.9, 36.2, and 62.5% respectively (*p* < 0.05). In contrast, the levels of SFAs, MUFAs, PUFAs, and total FAs in the PF group are reduced compared to the HF group, with respective decreases of 28.1, 25.2, 12.5, and 27.1%. Nonetheless, when compared to the HF group, the contents of SFAs and total FAs in the feces of mice in the PM group have increased by 28.0 and 15.1% respectively. Thus, it can be inferred that PF and PM have distinct impacts on lipid absorption and metabolism balance.

### Fecal Sterols

**Figure 9:**
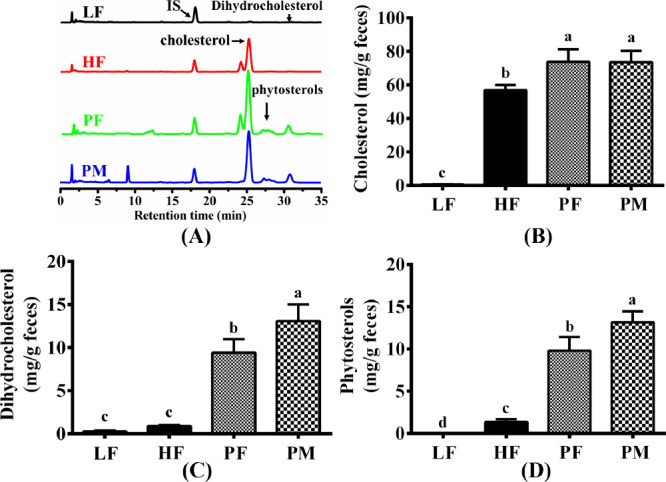
High-performance liquid chromatograms of fecal sterols (A) in four groups of mice and their contents of cholesterol (B), dihydrocholesterol (C), and phytosterols (D). LF: low-fat group (*n* = 8); HF: high-fat group (*n* = 7); PF: phytosterol ferulate group (*n* = 8); PM: physical mixture of phytosterols and ferulic acid group (*n* = 8); IS: internal standard. Data are expressed as the means with different superscript letters (a, b, c, and d) that differ significantly at *p* < 0.05.

Figure 9A displays the high-performance liquid chromatography profiles of sterols in the feces of mice in each group. In this graph, cholesterol and IS (ergosterol) were eluted at 25 and 18 min, respectively. Meanwhile, the retention times for phytosterols and dihydrocholesterol were 27 and 31 min, respectively. The fecal cholesterol levels of mice in each group are presented in Figure 9B. The LF group exhibited a significantly lower fecal cholesterol content than the other three groups, attributed to their consumption of a basal diet without added cholesterol. When compared to the HF group, the fecal cholesterol contents in both the PF and PM groups experienced significant increases of 29.7 and 29.2%, respectively, with no significant difference between the two. This suggests that both PF and PM can facilitate cholesterol excretion. Dihydrocholesterol is the hydrogenated product of cholesterol, resulting from the action of intestinal bacteria. As depicted in Figure 9C, the fecal dihydrocholesterol contents increase 9.5- and 13.6-fold in the PF and PM groups, respectively, compared to the HF group. As shown in Figure 9D, the content of phytosterols in the feces of both LF and HF groups was quite low, while that of PF and PM groups exceeded 10 mg/g. Although the content of phytosterols in PF and PM is identical, the latter has a 25.6% higher content of phytosterols in feces, suggesting that PF might be more easily absorbed by the body than PM.

## Discussion

Ferulic acid esters of phytosterols and triterpene alcohols together compose oryzanol, a group of biologically active compounds derived from rice bran or rice bran oil. PF accounts for less than 25% of the content in oryzanol.^11^ A prior study suggests that the cholesterol-lowering effect of rice bran oil containing oryzanol in the general population is likely attributed to phytosterols, rather than triterpene alcohols,^32^ indicating that PF might be the active components. Despite rice bran or its related products like rice bran oil and oryzanol being natural sources of PF, their natural quantity is low, making it impossible to obtain them in a large amount through the process of separation and extraction. To date, various methods have been employed to prepare/synthesize PF, including multistep chemical reactions, chemo-enzymatic catalysis, and enzymatic catalysis.^12−14^ However, these methods generally have some drawbacks, such as complexity, low conversion rates, or high costs, rendering them less than ideal for efficient preparation for PF. Therefore, the first objective of this study was to develop an efficient method for preparing PF using acidic ILs as a catalyst, with phytosterols and ferulic acid as substrates. Through the screening of ILs and optimization of reaction parameters (Figure 4), the optimal synthesis process of PF was established. Under these conditions, the conversion rate of phytosterols could reach over 99% after just 2 h of reactions (Figure 4). Although there were some byproducts, the selectivity of the target product PF was over 83% (Figure 4). By amplifying the optimal parameters 40 times, we could obtain gram-level PF. This study thus offered several advantages of simple operation, a high conversion rate, and a short reaction time, effectively addressing the shortcomings of existing technologies.

A previous study has suggested that oryzanol containing PF is a lipid-lowering factor.^22^ However, research on the lipid-lowering activity of PF is scarce. It remains unclear whether it is the active component in oryzanol. Therefore, a secondary objective of this study was to investigate the role of PF in alleviating dyslipidemia induced by feeding a high-fat diet in mice. Hypercholesterolemia, manifested primarily by elevated plasma TC and LDL cholesterol, is a risk factor for atherosclerosis and cardiovascular disease.^2^ In this study, both plasma TC and non-HDL-C were significantly increased in C57BL/6J mice after 15 weeks of feeding a high-fat diet, demonstrating the successful establishment of a high hypercholesterolemia model. Addition of 0.5% PF significantly mitigated the cholesterol metabolism disorder, primarily evidenced by reducing 13.7% plasma TC and 46.3% plasma non-HDL-C (Figure 5), thereby confirming that PF was the active ingredient or at least one of the active ingredients in oryzanol in ameliorating hypercholesterolemia. In line with our findings, feeding 0.5% oryzanol to hypercholesterolemic hamsters and rats notably reduced their plasma cholesterol levels.^33−35^ Moreover, we observed that, in comparison to HF, PF feeding led to a 55.8% reduction in liver cholesterol in mice (Figure 7). A previous study has suggested that oryzanol may reduce plasma cholesterol via inhibiting exogenous cholesterol absorption as such a hypocholesterolemic effect was abolished when oryzanol was added to a cholesterol-free diet.^36^ Our results indicate that the addition of 0.5% PF significantly increased the amount of cholesterol in feces (Figure 9B). Dihydrocholesterol is a metabolite of cholesterol produced in the colon under the action of intestinal microbiota,^37^ and its content significantly increased due to the supplementation of 0.5% PF (Figure 9C). Based on these analyses, PF is likely to achieve cholesterol-lowering efficacy by inhibiting cholesterol absorption.

Hypertriglyceridemia is also a key factor in causing obesity and nonalcoholic fatty liver diseases. In this study, after 15 weeks of high-fat diet-feeding C57BL/6J mice, their plasma TG levels substantially increased. However, PF significantly reduced plasma TG levels by 16.9% (Figure 5). This is consistent with previous studies that demonstrate that oryzanol and phytosterols can alleviate the increase in plasma TG levels induced by feeding high-fat diets.^21,38^ Moreover, a high-fat diet led to significant increases in weight gain, perirenal fat, and epididymal fat in mice. PF reversed these changes significantly, as demonstrated by a decrease in weight gain, perirenal fat, and epididymal fat by 20.9, 46.9, and 37.1%, respectively (Table 2). The results were in agreement with those of Wang et al., who found that oral administration of 3 mg/kg oryzanol significantly reduced weight gain and fat accumulation.^21^ The liver, being a crucial organ for lipid metabolism, exhibited a significant increase in total lipids and a significant accumulation of lipid droplets due to the high-fat diet, indicating the development of nonalcoholic fatty liver. However, PF reduced the total fatty acid content in the liver by 36.3% and effectively inhibited hepatic steatosis (Figure 6). Similarly, oryzanol, stigmasterol, and β-sitosterol have been proven to effectively alleviate high-fat diet-induced nonalcoholic fatty liver.^39,40^ These results confirm the significant potential of PF in preventing lipid accumulation, although the exact mechanism remains unclear. It is widely recognized that the absorption of exogenous lipids and endogenous synthesis are the two main sources of body lipids.^2^ In this study, PF significantly reduced the contents of fecal SFA, MUFA, and total FAs (Figure 8). These findings suggest that PF is most unlikely to lower body lipid levels by inhibiting the absorption of exogenous dietary lipids. It is worth noting that fatty acid synthase (FASN), a crucial enzyme in endogenous fatty acid synthesis, is regulated by liver X receptor alpha (LXRα).^2^ In the case of oleic acid-induced HepG2 cells, oryzanol significantly inhibited the expression of both FASN and LXRα.^34^ Based on these analyses, it is likely that PF reduces the lipid content in vivo by inhibiting endogenous lipid synthesis.

Additionally, this study also compared the hypolipidemic differences between PF and their substrates (a physical mixture of phytosterols and ferulic acid). A previous study has indicated that oryzanol releases triterpenoids (or sterols) and ferulic acid during intestinal digestion, both of which possess their own lipid-lowering properties.^41^ Similarly, the hydrolysis of PF by intestinal digestive enzymes could yield phytosterols and ferulic acid. Hence, it can be inferred that PF and PM feedings should exhibit the same lipid-lowering activity. However, our findings contradict this inference as we observed no lipid-lowering effects from PM (Figure 5). This may be related to the extent of hydrolysis of PF during intestinal digestion. In this study, the phytosterol equivalents in the PF and PM diets were the same, but their contents in the feces differed. It is well known that the concentration of test substances in feces can largely reflect their absorption. In particular, the fecal content of phytosterols in the PF group exhibits a 25.6% reduction compared to the PM group (Figure 9), suggesting that PF was more readily absorbed in the small intestine than PM. A previous study has revealed that when rabbits were orally administered ^14^C-labeled oryzanol, 80% of the radioactivity in the blood originated from ferulic acid,^42^ implying that oryzanol undergoes hydrolysis into ferulic acid and exists within the body. More recently, intact oryzanol has been detected in the plasma of mice orally administered with oryzanol,^43^ and a higher oryzanol content in the plasma correlates with lower blood lipid levels.^44^ This suggested that intact oryzanol possessed better lipid-lowering activity.

Hypocholesterolemic activity of phytosterols has been widely recognized. However, this study did not observe such activity in PM. This discrepancy could be attributed to the different types of experimental animals used. The cholesterol-lowering activity of phytosterols has mostly been observed in hamsters^33^ or rats.^34^ Rideout et al. found that phytosterols did not reduce plasma TC and non-HDL-C levels when using C57BL/6J mice as the animal model.^38^ Additionally, although ferulic acid has shown cholesterol-lowering activity in vitro cell studies or rats, this effect was not observed in C57BL/6J mice.^45,46^ A previous study has shown that mice are less prone to developing hypercholesterolemia due to their higher expression of low-density lipoprotein receptor levels.^47^ Therefore, this could be one of the reasons why PM did not exhibit cholesterol-lowering activity. Triterpene alcohols and phytosterols extracted from rice bran (TASP) showed a dose-dependent effect on regulating plasma cholesterol in C57BL/6J mice. Supplementation with 0.04% TASP and 0.2% TASP for 23 weeks did not lower plasma TC levels, but 0.5 and 1% TASP reduced plasma TC by 19.8 and 23.4%, respectively.^48^ In this study, the content of phytosterols in the PM group was 0.3%, suggesting that the dosage could also be one of the factors influencing the cholesterol-lowering activity of phytosterols.

In conclusion, we successfully and efficiently synthesized PF for the first time using [BSO_3_HMim]OTF as a catalyst. Our findings demonstrated that the dietary consumption of 0.5% PF could significantly alleviate hyperlipidemia, as evidenced by significant reductions in plasma and hepatic lipids. Notably, the lipid-lowering activity of PF surpassed that of its precursors, PM. However, the specific mechanism by which PF exerts its hypolipidemic effects remains unclear and warrants further in-depth studies in the future. It is concluded that PF has an exceptional lipid-lowering activity and has great potential as a dietary ingredient in management of dyslipidemia.
